# Characterizing industry payments for FDA-approved AI medical devices

**DOI:** 10.1093/haschl/qxaf211

**Published:** 2025-11-10

**Authors:** Alon Bergman, Tej A Patel, Kaustav P Shah

**Affiliations:** Department of Medical Ethics and Health Policy, University of Pennsylvania, Philadelphia, PA 19104, United States; Department of Healthcare Management, The Wharton School, University of Pennsylvania, Philadelphia, PA 19104, United States; Leonard Davis Institute of Health Economics, Philadelphia, PA 19104, United States; The Wharton School, University of Pennsylvania, Philadelphia, PA 19104, United States; Nuffield Department of Population Health, University of Oxford, Oxford, OX3, 7LF, United Kingdom; Leonard Davis Institute of Health Economics, Philadelphia, PA 19104, United States; Department of Medicine, University of Pennsylvania, Philadelphia, PA 19104, United States; Corporal Michael J. Crescenz Medical Center, Veterans Affairs, Philadelphia, PA 19104, United States

**Keywords:** medical devices, artificial intelligence, open payments, physicians, conflicts, digital divide

## Abstract

**Introduction:**

Artificial intelligence-enabled medical devices (AIMDs) are increasing in use, but this growth has raised concerns about inequities in access across well-resourced and under-resourced settings. Little is known about industry–clinician partnerships in the AIMD ecosystem.

**Methods:**

We examined the value, specialty distribution, market concentration, and institutional profile of payments made by industry to clinicians for Food and Drug Administration-approved AIMDs using the Open Payments Database. We linked payments to the affiliated hospital of the clinician using the Medicare Provider Data catalog. We performed a regression to explain the association of payments with hospital and county-level factors.

**Results:**

We found $59.3 million was spent on payments to 46 315 clinicians for AIMDs between 2017 and 2023, representing an increasing share of total medical device payments over time. We saw high payment concentration in technologically intensive medical specialties and among clinicians affiliated with large, urban teaching hospitals.

**Conclusion:**

Industry payments for AIMDs are increasing and concentrated among technology-intensive specialties. Payments are more likely to flow to clinicians affiliated with teaching hospitals that are larger and in non-rural areas. This may reflect or mediate increased AI utilization in these settings. Continued monitoring of payments, transparent reporting, and targeted resource support may be needed to promote equitable access to AIMDs.

## Introduction

The use of artificial intelligence (AI) in medical devices has experienced unprecedented growth, with Food and Drug Administration (FDA) clearances increasing by 350% in the past 5 years.^1^ As of July 2025, over 1200 AI-enabled medical devices (AIMDs) have received FDA approval, spanning specialties from radiology to cardiology to neurology.^1^ AIMDs range from software that interprets medical imaging, such as automated identification of lung abnormalities on chest X-rays to AI-embedded hardware, including intraoperative imaging systems that assist surgeons in real time and portable devices that support bedside evaluation of neurological or cardiac function. Currently, the majority of FDA-approved AIMDs use deep learning architecture, and there are no approved devices that use large language models or other forms of generative AI.^2^

This rapid expansion in regulatory clearances has paralleled significant advances in AI adoption across these clinical areas.^3-14^ However, this growth has not reached all healthcare settings evenly.^15-23^ Recent studies demonstrate that hospitals serving rural communities, those without teaching affiliations, and facilities in lower-income areas show reduced utilization of AI both for medical devices^24^ and administrative tasks.^25^ Survey data reveal that similar inequities exist in hospital adoption of AI predictive models.^26,27^ These disparities mirror historical patterns with electronic health records and other digital technologies.^28,29^

Although the mechanisms reinforcing this AI digital divide are still being explored, one potential explanation is the relationship between industry partners and clinicians, which may play an outsized role in shaping which technologies are used, by whom, and in what settings. Industry payments to clinicians have long influenced the diffusion of medical technologies, especially in device-heavy specialties such as cardiology and neurosurgery.^30-33^ The Open Payments program, mandated by the Affordable Care Act, has made financial relationships between device vendors and clinicians observable to researchers and the public. Previous research has established strong associations between device manufacturer payments and physicians’ selection of specific implants, with physicians being substantially more likely to use devices from manufacturers providing them with payments.^30-32^ Medical device firms allocate approximately 1.7% of industry revenue to physician payments—7 times the percentage spent by pharmaceutical companies—with payments concentrated among surgical specialists and often related to training and product development.^33^

It remains unclear whether the financial relationships underpinning traditional devices extend to the emerging AIMD ecosystem and whether such relationships exacerbate technology access disparities. The regulatory landscape for AIMDs adds another layer of complexity to understanding adoption patterns. The majority of AIMDs have been cleared through the FDA's 510(k) pathway, which requires only demonstrating substantial equivalence to previously approved devices. These “predicate” devices often lack AI components and were approved for different tasks than the AIMDs using them as benchmarks.^34^ This regulatory ambiguity may increase the influence of industry relationships, as health systems lacking robust internal evaluation capabilities and uncertain of the regulatory rigor required for approval may rely more heavily on vendor partnerships and clinician champions when making adoption decisions.^35^

This study presents the first comprehensive analysis of industry payments related to AIMDs. First, we analyzed AIMD payment trends from 2017 to 2023 and characterized AIMD payments by medical specialty as a share of overall medical device payments. Next, we assessed the degree of AIMD market concentration among medical specialties. Finally, we identified hospital and county-level characteristics associated with affiliation of clinicians who received AIMD payments.

## Methods

### Data and sample

We used data from 2017 to 2023 (the most recent available year) from the Centers for Medicare & Medicaid Services (CMS) Open Payments database. Open Payments, established under the Physician Payments Sunshine Act, records financial transfers of value from pharmaceutical, medical device, and biologics manufacturers to clinicians and teaching hospitals in the United States. The database includes both research and non-research payments and captures details such as the name of the entity making the payment and the recipient of the payments, as well as the amount, date, and nature of payment (eg, consulting fees, meals, travel, speaker compensation, or ownership interests). We focused on non-research (general) payments, consistent with prior literature, as these represent direct financial relationships with individual clinicians and differ substantially in nature from research payments.^33,36,37^

We limited our analysis to payments made by medical device firms. We defined a firm as a “medical device firm” if 90% or more of the payments it made to clinicians, in terms of value, were affiliated with a medical device. (A similar approach was taken by Bergman (2021) in classifying firms appearing in Open Payments data to pharmaceutical and medical device firms.)

We derived data on AIMDs from the FDA AI/ML-enabled Medical Devices List, which reports on AI/ML-enabled devices authorized for marketing in the United States. The list contains the FDA submission number, device name, company name, product code, and FDA panel. We manually matched FDA products and firm names to the Open Payments dataset. Firm and device names were standardized (eg, removal of non-alphabetic characters, conversion to lowercase, and stripping of common corporate suffixes), and matches were based on substring searches of firm and device names cross-referenced for internal consistency. To assess validity, a random 10% sample audit of FDA-listed devices found 78% of device matches and 89% of payment value were correctly captured in our dataset. For firms that market both AIMDs and other devices (eg, Medtronic), we only included payments affiliated with AIMDs by manually matching products in the FDA AI/ML-enabled devices list to Open Payments products. For firms that only market AIMDs (eg, Aidoc Medical), we fully attributed payments to AIMDs, following manual review of company websites to confirm exclusive marketing of AIMDs.

After manual review, we matched 95 of the 390 firms (24.4%) in the FDA AI/ML-enabled devices list to firms in the Open Payments database. The relatively low match rate reflects that many FDA-listed device manufacturers made no reportable payments to clinicians during the study period.

We matched clinicians in the Open Payments dataset to the 2017-2023 Medicare Provider Data Catalog (PDC) based on National Provider Identifier (NPI). The PDC contains information on Medicare-accepting clinicians’ hospital affiliations and specialties. Overall, we successfully matched 89.5% of NPIs receiving AIMD payments to the PDC. The match allowed us to identify the primary hospital affiliation for each clinician. (Medicare PDC lists up to 5 hospital affiliations for each clinician and year. We define a clinician's primary hospital as their first reported hospital. Clinicians’ hospitals affiliations are not ordered in 2023, for the 25.7% of clinicians who have more than one hospital affiliation. For that year, we order each clinician's affiliations based on their observed affiliation order in 2022, when available, and by ascending order when unavailable). The dataset further allowed us to calculate the total number of Medicare clinicians in each specialty and for each given year. Non-physician healthcare professionals were included in the aggregate payment trend analysis to reflect total AIMD payment volume but were excluded from all specialty- and hospital-level analyses, which were restricted to physicians (MDs/DOs) matched to the Medicare Provider Data Catalog.

Finally, we used the American Hospital Association (AHA) Annual Survey to retrieve information about hospitals’ size, location, ownership status, geography, patient mix, and financial performance. We obtained additional information on hospital resources by linking the AHA Supplement with the Medicare Cost Report to hospital operating margins and uncompensated care burden. We used the American Community Survey to obtain county-level income and social deprivation index for each hospital. To compare the county-level and hospital characteristics of hospitals whose clinicians received AI payments, we linked data from the Open Payments Website to the AHA Survey using each observation's associated CMS certification number.

### Outcomes

Our primary outcomes were the share of clinicians receiving AIMD-related payments (the payment “rate”) and the total dollar value of these payments (the payment “magnitude”). We examined these outcomes both in aggregate and disaggregated by specialty, device manufacturer, and hospital affiliation characteristics. For calculating the share of clinicians in a given specialty receiving payments, we included only clinicians with NPIs present in both the PDC and Open Payments databases and excluded any payments that could not be attributed to a specific medical specialty. Specifically, the share of clinicians receiving either AIMD or general medical device payments in any given specialty was calculated by dividing the number of distinct clinicians who received payments across the study period (identified by unique NPIs) by the total number of distinct clinicians in that specialty documented in the PDC.

In subsequent analyses of the AHA survey data, we also assessed geographic and institutional variation in payment receipt, specifically analyzing the probability that a hospital had at least one affiliated clinician receiving an AIMD-related payment as a function of clinician and hospital characteristics (payment “targeting”) using multivariate Poisson regression. The predictors used in this analysis included hospital size (small: <100 beds; medium: 100-399 beds; and large: ≥400 beds), teaching status (nonteaching hospital and academic medical center), ownership status (for-profit, nonprofit, and government [state, county, or city] owned), health system membership, rural status, and critical hospital status. Hospital operating margins, uncompensated care burden, county-level income, and the hospital’s Social Deprivation Index based on primary zip code were included as operational and county-level factors affecting AI adoption.

## Results

### Payment volume and types

**Figure 1.**
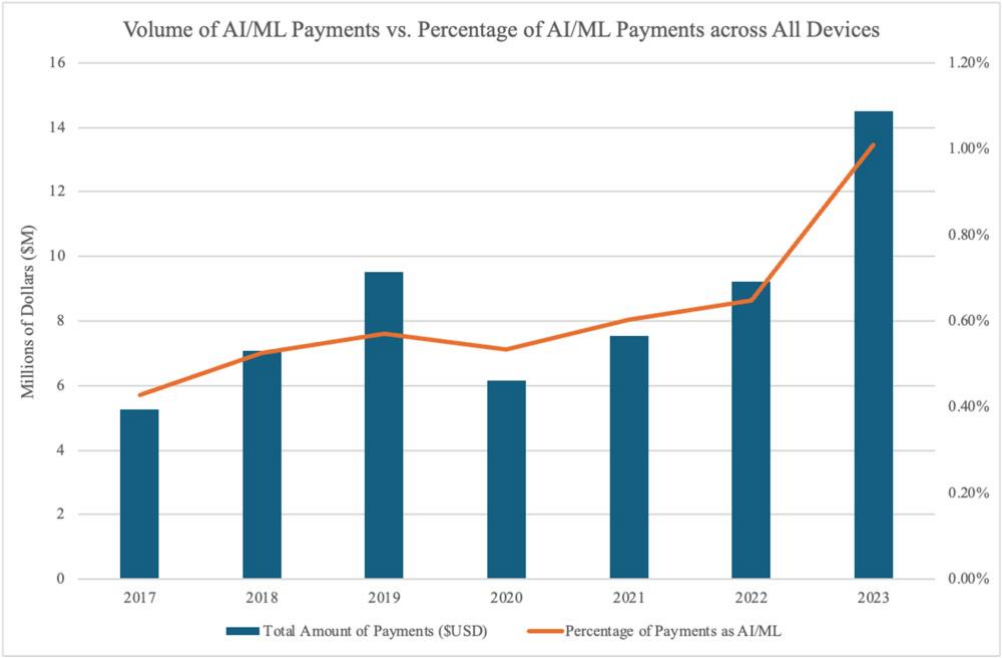
Annual AIMD payment volume and share of all device payments, 2017-2023. SOURCE Authors’ analysis of Open Payments data from 2017-2023. NOTES Volume of total AIMD payments to all clinicians is graphed in the bar plot by year, whereas the percentage of AIMD payments as a share of total device payments is represented in the accompanying line graph. AIMD = artificial intelligence-enabled medical device.

We present the first analysis of AIMD industry payments from the Open Payments database and find substantial growth in the magnitude of non-research AIMD payments made to clinicians from approximately $5.3 million in 2017 to $14.5 million in 2023, with particularly sharp growth observed in the final year of the study period (Figure 1). The total payment volume for AIMDs was $59.3 million with payments made to 46 315 clinicians. Over the same span, the share of general device-related payments attributable to AIMDs among medical devices overall increased from 0.43% in 2017 to 1.01% in 2023.

The distribution of AIMD payments by payment type (eg, consulting fees, speaker compensation, meals, travel) closely mirrored that of general medical device payments, with 96.9% of overall clinicians receiving payment for food and beverage (Appendix Table S1).

### Payments by subspecialty and vendor

From 2017 to 2023, the specialties receiving the largest total AIMD payment amounts were neurological surgery ($7 664 569), cardiac electrophysiology ($7 332 643), cardiology ($6 642 962), and diagnostic radiology ($5 125 721) (Table 1). Respectively, these specialties ranked #2 (neurosurgery: $550 026 891), #10 (cardiac electrophysiology: $138 230 908), #4 (cardiology: $244 053 806), and #7 (diagnostic radiology: $154 301 208) in overall device payments during this period.

**Table 1.** Payment rates and amounts for AIMD and general medical device payments by specialty.

Specialty	AIMDs				All medical devices			
	Total payment value ($1000)	Medicare clinicians receiving payments (%)	Average payment volume ($)		Total payment value ($1000)	Medicare clinicians receiving payments (%)	Average payment volume ($)	
			Average	P10, P50, P90			Average	P10, P50, P90
Neurological surgery	7665	30.9	703	(1 1, 65, 684)	550 027	88.6	1580	(1 1, 51, 843)
Cardiac electrophysiology	7333	74.9	156	(5, 21, 124)	138 231	90.2	323	(7, 30, 450)
Cardiology	6643	34.4	136	(8, 21, 125)	244 054	73.0	240	(9, 23, 182)
Diagnostic radiology	5126	7.6	517	(1 2, 48 , 307)	154 301	44.4	596	(1 2, 39, 500)
Orthopedic surgery	5086	5.9	556	(1 1, 66 , 627)	3 298 909	84.2	2720	(14, 86, 1 584)
Neurology	2003	6.2	664	(1 2, 28, 204)	42 063	55.7	303	(1 2, 26, 328)
Emergency medicine	1694	1.0	2298	(15 , 471, 1 616)	17 352	22.5	405	(1 2, 30, 343)
Interventional cardiology	1287	48.6	116	(1 0, 23, 142)	148 288	86.5	297	(9, 27, 289)
General surgery	1151	2.1	764	(14, 75, 1 000)	298 254	76.2	494	(13, 60, 1 000)
Radiation oncology	1028	13.2	472	(1 4, 64 , 521)	41 814	74.8	1027	(1 4, 54, 800)
Internal medicine	732	2.1	85	(1 0, 23, 125)	59 859	35.2	159	(1 2, 21, 137)
Gastroenterology	622	1.2	1401	(15, 67, 2 125)	62 094	69.3	522	(1 2, 50, 503)
Obstetrics & gynecology	357	0.9	419	(1 4, 63, 604)	116 483	67.4	339	(1 2, 22 , 269)
Ophthalmology	342	0.2	4278	(14, 47, 19 230)	181 384	76.7	568	(1 2, 26, 191)
Advanced heart failure and transplant cardiology	321	18.6	691	(1 3, 29, 230)	14 982	87.5	388	(1 3, 59, 701)

Source: Authors’ analysis of Open Payments data from 2017 to 2023.

Total payment by specialty (Medicare clinicians), percentage of Medicare clinicians receiving payment, and mean device payments, stratified across AIMDs and all medical devices. The top 15 clinical specialties, by artificial intelligence payment volume, are represented in descending order. Note average payment represents the average amount in a single payment (eg, from vendor to clinician), not the average payment per clinician.

Abbreviation: AIMD = artificial intelligence-enabled medical device.

Among Medicare-accepting clinicians, cardiac electrophysiology (74.9%), interventional cardiology (48.6%), general cardiology (34.4%), and neurological surgery (30.9%) had the highest share of clinicians receiving AIMD-related payments. These specialties also tended to have among the highest share of clinicians receiving any general device-related payments: for example, 90.2% of cardiac electrophysiologists and 88.6% of neurological surgeons participating in the Medicare program received a general device payment across the study period. This relationship was not always present; for example, general surgery had a low proportion of clinicians receiving AIMD payments (2.1%) despite high overall device payment rates (76.2%). Most specialties had single-digit percentages of clinicians receiving AIMD payments.

Average payment amounts varied by specialty. Within several specialties, the standard deviation in payment size was large, indicating high dispersion in payment magnitude among recipients. For example, in emergency medicine, the average payment was $2,298, but payments ranged widely, with a standard deviation of $12 694. On average, AIMD-related payments were smaller in magnitude than overall medical device payments.

Within each specialty, payments were made by a small number of firms. Table 2 presents the total value of AIMD-related payments from leading medical device firms and their respective market shares within key medical specialties. Overall, the 10 highest-paying firms accounted for approximately 64% of all AIMD-related device payments made to clinicians. Payments were concentrated in specific specialty markets, with single firms frequently dominating payments within particular specialties. For example, Biosense Webster accounted for 82.5% of payments to cardiac electrophysiology, and Ceribell accounted for 69.1% of payments in neurology. Medtronic was notably prominent across multiple specialties, including neurosurgery (23.1% share) and orthopedic surgery (18.5% share). Across specialties, most top-paying firms focused their payments within 1 or 2 areas, and payment shares varied widely between specialties.

**Table 2.** Top-paying device vendors’ total and share of AI payments by clinical specialty panel.

Firm	AIMD payment total ($1000)	Share of all AIMD payments in given specialty (%)							
		Card.	Card electro.	Int. card.	Neuro.	Neurosurg.	Rad.	Orthopedic surg.	E.M.
Biosense Webster	8982	20.0	82.5	5.1	0.1	0.0	0.1	0.0	0.7
Medtronic	5606	7.2	4.5	7.6	10.4	23.1	0.2	18.5	0.3
Nuvasive	5395	0.0	0.0	0.0	0.1	43.4	0.0	38.5	0.1
Irhythm Technologies	3250	22.3	6.0	3.0	0.1	0.0	0.0	0.0	6.7
Siemens	3005	1.8	0.3	0.6	0.7	0.4	33.1	0.1	0.1
Heartflow	2589	21.5	0.2	22.5	0.0	0.0	6.1	0.0	0.4
Ceribell	2543	0.0	0.0	0.0	69.1	0.9	0.0	0.0	15.9
Brainlab	2426	0.0	0.0	0.0	0.2	5.7	0.0	9.5	0.0
Clearpoint Neuro	2039	0.0	0.0	0.0	0.0	8.2	0.0	0.0	0.0
Perspectum Diagnostics	1955	0.0	0.0	0.0	0.0	0.0	16.6	0.0	2.2
Total from top 10 Paying firms	37 791	73.0	93.0	39.0	81.0	82.0	56.0	67.0	26.0

Source: Authors’ analysis of Open Payments data from 2017 to 2023.

Abbreviations: AIMD = artificial intelligence-enabled medical device; Cardio. = cardiovascular; Card Electro. = cardiac electrophysiology; Int Card. = interventional cardiology; Neuro. = neurology; Neurosurg. = neurosurgery; Rad. = radiology; Orthopedic Surg. = orthopedic surgery; E.M. = emergency medicine.

### Classifying payments by affiliated hospital

Finally, we assessed the relative risk ratios (RRs) from a multivariable regression analysis evaluating hospital characteristics associated with the receipt of AIMD-related payments by affiliated clinicians (Table 3). Larger hospitals (≥400 beds) had significantly higher likelihoods of associated clinician payments (RR 1.76; 95% CI: 1.51-2.04) compared to small hospitals (<100 beds). Teaching hospitals were also significantly more likely to have affiliated clinicians receiving AIMD-related payments compared to non-teaching hospitals (RR 1.34; 95% CI: 1.21-1.47). Non-rural hospitals had a higher likelihood of payment (RR: 1.31, 95% CI: 1.16-1.48) compared to rural hospitals. Government-owned hospitals (RR 0.73; 95% CI: 0.65-0.83) and for-profit hospitals (RR 0.73; 95% CI: 0.65-0.82) had lower payment probabilities relative to non-profit hospitals. Higher social deprivation scores were associated with lower payment probabilities (RR 0.86; 95% CI: 0.75-0.98 for the highest deprivation quartile). Finally, non-critical access hospitals (RR 2.08; 95% CI: 1.76-2.44) had notably higher likelihoods of payments.

**Table 3.** Adjusted risk of receiving AI payments by hospital characteristics.

Receiving payment from AIMD manufacturer	Relative risk ratio (95% CI)	*P* value
Hospital size (Ref.: <100 beds)		
Medium (100-399 beds)	1.72 (1.54-1.92)	<.001
Large (≥400 beds)	1.76 (1.51-2.04)	<.001
Teaching status (Ref.: non-teaching)		
Teaching	1.34 (1.21-1.47)	<.001
Ownership (Ref.: non-profit)		
For-profit	0.73 (0.65-0.82)	<.001
Government	0.73 (0.65-0.83)	<.001
Health system membership (Ref.: non-member)		
Member	1.10 (1.00-1.21)	.05
Region (Ref.: Northeast)		
South	1.09 (0.96-1.24)	.18
Midwest	1.06 (0.93-1.20)	.42
West	1.19 (1.04-1.36)	.01
Rural status (Ref.: Rural)		
Non-rural	1.31 (1.16-1.48)	<.001
Median household income (Ref.: <$45 000)		
$45 000–$59 999	1.25 (0.93-1.67)	.13
$60 000–$79 999	1.31 (0.97-1.77)	.08
≥80 000	1.23 (0.89-1.70)	.21
Social Deprivation Index (Ref.: <25)		
25-49	0.96 (0.85-1.08)	.47
50-74	0.89 (0.79-1.01)	.07
≥75	0.86 (0.75-0.98)	.03
Critical access hospital (Ref.: critical access hospital)		
Non-critical access hospital	2.07 (1.76-2.44)	<.001
Constant	0.11 (0.08-0.16)	<.001

Source: Authors’ analysis of America Hospital Association (AHA) Survey data.

Multivariate Poisson regression models with a binary outcome and log-linear link were used to generate relative risk ratios and 95% CIs to predict hospital-level factors associated with AIMD-payment receipt. Clinician-level data from Open Payments were linked with the corresponding hospital CMS certification number from the AHA Survey.

Abbreviations: AI = artificial intelligence; AIMD = artificial intelligence-enabled medical device.

## Discussion

We present a comprehensive analysis of non-research industry payments for FDA-approved AIMDs and find $59.3 million of AIMD payments were made from 2017 to 2023 and were concentrated among select clinicians, firms, and institutions. During this period, annual AIMD-related payments increased from $5.3 million to $14.5 million, with their share of total device payments more than doubling from 0.43% to 1.01%. These payments were concentrated in technology-intensive specialties, including neurological surgery, cardiology, and associated subspecialties, including cardiac electrophysiology, interventional cardiology, and diagnostic radiology. Payments were associated with clinicians affiliated with large, teaching hospitals in urban, high-income areas. The market for AIMD-related payments showed high concentration, with single firms accounting for over 60% of payments in specialties such as cardiac electrophysiology and neurology. Our findings show a commercial architecture surrounding AIMDs that mirrors the longstanding concerns about financial influence in device adoption. Yet unlike traditional medical technologies, AIMDs carry unique risks due to opaque device performance, limited oversight, and uneven real-world evidence.^19-23^

The growth in AIMD-related clinician payments reflects broader trends in medical technology innovation and commercialization. The nearly 350% increase in FDA clearances of AI devices over the past 5 years represents a fundamental shift in the medical device landscape. Our findings suggest that AIMD manufacturers are investing heavily in clinician engagement. Investment patterns parallel historical precedents in medical device markets, where close clinician–industry collaboration has been essential for both product refinement and clinical adoption.^33,38^ Many technology-intensive specialties like neurosurgery, cardiology, and radiology appear to be at the forefront of AIMD investment; however, this relationship did not always hold. General surgery, for example, had 2.1% of Medicare-participating clinicians receiving payments despite being a procedural and device-intensive field with high general medical device payments (76.2%) and 6 approved AIMDs by the FDA General and Plastic Surgery panel. Further work should explore the mechanisms of these differences by specialty.

The concentration of payments in teaching hospitals and urban centers may contribute to disparities in AI technology access between urban and rural settings and between academic and non-academic institutions. Our results align with prior evidence documenting lower AI utilization in rural and non-teaching settings.^24,25^ Industry payments both reflect and potentially mediate this digital divide. Teaching hospitals are affiliated with clinicians who have research expertise, professional society leadership roles, and educational responsibilities that often lead to industry partnerships. These institutions also possess the technical infrastructure and financial resources necessary to evaluate and implement novel technologies. In contrast, rural and smaller hospitals may lack both the clinical volume to attract industry attention and the organizational capacity to assess emerging AI technologies.^26^ Future work should rigorously evaluate causal relationships between industry payments and hospital utilization of AIMDs through claims data, although few AIMDs currently have reimbursement mechanisms.

The high market concentration in several specialties raises competitive concerns. When single firms account for more than 80% of payments within a specialty, as seen with Biosense Webster in cardiac electrophysiology, this dominance may reflect successful market positioning by established vendors or aggressive market entry by new competitors. However, such concentration raises concerns similar to those documented in traditional medical device markets, where limited competition has been associated with higher prices, reduced innovation incentives, and supply chain vulnerabilities.^39^

Our study had several limitations. First, our analysis of payment percentage by specialty was limited to clinicians participating in Medicare and, therefore, may not fully capture payment patterns among all clinicians. Additionally, 14.7% of payment dollars could not be attributed to a medical specialty. Second, we relied on the FDA's AI/ML-enabled medical device list to obtain relevant devices, which is not a comprehensive inventory of all devices incorporating AI. As the FDA notes, the list is primarily based on information disclosed in marketing authorization summaries and may under- or over-represent the true scope of AI-enabled technologies currently in use. Many of the most widely implemented healthcare AI solutions using generative AI, such as ambient scribes, have not been regulated by the FDA. Third, our analysis did not include clinician-level data on actual device utilization or clinical adoption, limiting our ability to assess whether industry payments influence practice behavior or downstream patient outcomes. Importantly, we do not interpret the absence of payments for most FDA-approved AIMD manufacturers as evidence of underreporting. Ambiguity in reporting requirements primarily affects unregulated software vendors that do not meet the FDA definition of a medical device, but not manufacturers of AIMDs who have explicitly sought FDA authorization for a regulated medical device and are therefore subject to the Physician Payments Sunshine Act reporting requirements. As such, we interpret the lack of payments recorded for most AIMD manufacturers likely reflects the true absence of financial relationships with clinicians, rather than omissions or gaps in reporting. Fourth, as Open Payments does not consistently identify product-level associations, our approach—fully attributing payments from AIMD-only firms and limiting attribution for multiproduct firms to explicit AIMD-related payments—may introduce minor asymmetry, likely biasing results toward underestimating total AIMD-related payment volumes. Finally, our primary analysis focused on non-research payments, consistent with prior literature examining physician–industry financial relationships. Research-related payments, which often flow to institutions rather than individual clinicians, totaled approximately $60 million for AIMDs during the study period. Including these payments does not meaningfully alter hospital-level associations but may represent an additional channel of industry engagement concentrated in large academic centers.

Our findings have implications for monitoring AI device adoption patterns and speak to the importance of transparent financial reporting through the Open Payments program. The concentration of industry payments among clinicians at large teaching hospitals suggests that current industry engagement strategies may reinforce existing disparities in technology access. Specialties with high market concentration in payments, like cardiac electrophysiology, should be further studied to see if payment consolidation represents true market consolidation for AIMDs. If the markets appear uncompetitive, this may disadvantage health systems that have limited negotiating power and ability to procure devices that meet true clinical needs. Finally, transparent reporting of conflicts of interest by clinicians allows the public, fellow clinicians, and other stakeholders to verify how financial ties may influence the development of clinical practice guidelines or local health system protocols that shape adoption and patient access.

The regulatory environment for AI devices adds complexity to these considerations. The predominant use of the 510(k) pathway, which requires only demonstration of substantial equivalence to predicate devices, may be insufficient for technologies that continuously learn and evolve.^35,40^ This regulatory uncertainty increases the importance of post-market surveillance and transparent reporting of industry relationships. Without robust clinical evidence, hospitals may heavily depend on vendor partnerships and clinical champions, which increases the influence of financial relationships on adoption decisions. Smaller and rural hospitals without these partnerships, who would typically rely on FDA guidance for drug or device adoption, may be more skeptical of AIMD usage, given the concerns about regulatory rigor.

Targeted interventions may be necessary to promote equitable access to AI innovations. First, programs modeled on the Regional Extension Centers that facilitated electronic health record adoption could help smaller and rural hospitals evaluate and utilize AIMDs through procurement advisory services, assistance with implementation and project management, and ongoing technical support and maintenance.^27^ Second, the FDA approval process could require more representative patient populations in clinical trials, particularly from non-academic and rural settings, and more technologically appropriate predicate comparisons for AIMDs to increase confidence in the regulatory approval process, potentially reducing the need for internal clinical champions with vendor relationships.^16,17^ Finally, as payment pathways for AIMDs are still being developed, payors could reimburse for devices that have been demonstrated to work effectively across multiple health settings with more diverse populations, creating financial incentives for adoption of high-quality AIMDs.^41^

## Conclusion

Industry payments for AI-enabled medical devices are growing rapidly and concentrated among clinicians at large, urban, teaching hospitals. These payment patterns may reflect or potentially contribute to existing disparities in AI technology adoption. Future work should rigorously investigate causal mechanisms linking AI industry payments to utilization of AIMDs. As AIMDs become more prevalent in clinical practice, continued monitoring of financial relationships between device manufacturers and clinicians will be essential for understanding their impact on equitable access to AI innovations.

## Supplementary Material

qxaf211_Supplementary_Data
