# Supplementary material for: Characterizing industry payments for FDA-approved AI medical devices
Source: Health Aff Sch. 2025 Nov 10;3(12):qxaf211. doi: 10.1093/haschl/qxaf211 (PMC12679402; doi:10.1093/haschl/qxaf211)
Supplement: qxaf211_Supplementary_Data [file qxaf211_supplementary_data.zip › HAS_Supplemental_Table_1.docx]

**Supplemental Table 1: AIMD payments to clinicians, stratified by type of payment**

| **Payment types** | **Total Amount of payment ($)** | **Clinicians, (n)** | **% Clinicians receiving payment type** | **Avg. payment amount ($)** |
| --- | --- | --- | --- | --- |
| Royalty, Licensing, and Investments | 7,645,349.19 | 59 | 0.1% | 129,582.19 |
| Consulting Fee | 26,579,235.64 | 1,593 | 3.4% | 16,685.02 |
| Speaking Fee | 4,741,568.69 | 564 | 1.2% | 8,407.04 |
| Travel and Lodging | 7,365,963.80 | 3,685 | 8.0% | 1,998.90 |
| Food and Beverage | 7,973,945.28 | 44,886 | 96.9% | 177.65 |
| Education | 708,491.42 | 124 | 0.3% | 5,713.64 |
| Other (Gifts, entertainment, and charitable contributions) | 228,948.95 | 181 | 0.4% | 1,264.91 |
| **TOTAL FROM 2017-2023** | 59,314,839.18 | 46,315 | –– | 1,280.68 |
